# High Incidence of HIV-1 Infection in a General Population of Fishing Communities around Lake Victoria, Uganda

**DOI:** 10.1371/journal.pone.0094932

**Published:** 2014-05-27

**Authors:** Noah Kiwanuka, Ali Ssetaala, Annet Nalutaaya, Juliet Mpendo, Matthias Wambuzi, Annet Nanvubya, Simon Sigirenda, Paul Kato Kitandwe, Leslie Elizabeth Nielsen, Apolo Balyegisawa, Pontiano Kaleebu, Josephine Nalusiba, Nelson Kaulukusi Sewankambo

**Affiliations:** 1 Makerere University College of Health Sciences, School of Public Health, Kampala, Uganda; 2 Uganda Virus Research Institute-International AIDS Vaccine Initiate HIV Vaccine Program, Entebbe, Uganda; 3 International AIDS Vaccine Initiative (IAVI), New York, New York, United States of America; 4 Medical Research Council/Uganda Virus Research Insitute, Uganda Research Unit on AIDS, Entebbe, Uganda; 5 Makerere University College of Health Sciences, School of Medicine, Clinical Epidemiology Unit, Kampala, Uganda; University of Pennsylvania School of Medicine, United States of America

## Abstract

**Background:**

High HIV-1 incidence rates were reported among persons in fisherfolk communities (FFC) in Uganda who were selected for high risk behaviour. We assessed the incidence of HIV-1 and associated risk factors in a general population FFC to determine population-wide HIV rates.

**Methods:**

A community-based cohort study was conducted among a random sample of 2191 participants aged 18–49 years. At baseline and 12 months post-baseline, data were collected on socio-demographic characteristics and risky behaviors (including number of partners, new partners, condom use, use of alcohol and illicit drug use). Venous blood was collected for HIV serological testing. HIV incidence was calculated per 100 person years at-risk (pyar) and adjusted incidence rate ratios (Adj.IRR) were estimated by multivariable Poisson regression.

**Results:**

Overall follow up at 12 months was 76.9% (1685/2191) and was significantly higher among HIV uninfected persons and those with at least 1 year duration of stay in community. Overall HIV-1 incidence was 3.39/100 pyar (95% CI: 2.55–4.49). Among the 25–29 years who drank alcohol, HIV incidence was 7.67/100pyar (95% CI;4.62–12.7) while it was 5.67/100pyar (95% CI;3.14–10.2) for 18–24 year olds who drank alcohol. The risk of HIV infection was higher among 25–29 years (adj.IRR = 3.36; 95% CI: 1.48–7.65) and 18–24 years (adj.IRR = 2.65; 95% CI: 1.05–6.70) relative to 30+ years. Compared to non-drinkers, HIV incidence increased by frequency of alcohol drinking - occasional drinkers (adj.IRR = 3.18; 95% CI: 1.18–8.57) and regular drinkers (adj.IRR = 4.93; 95% CI: 1.91–12.8).

**Conclusion:**

HIV-1 incidence in general fisherfolk population along L.Victoria, Uganda, is high and is mainly associated with young age and alcohol drinking. HIV prevention and control strategies are urgently needed in this population.

## Background

The HIV epidemic in sub-Saharan Africa is generalized and stable or declining [1]. High risk sub-groups can co-exist within generalized epidemics giving rise to concentrated HIV sub-epidemics in generalized epidemic settings [2]. These high risk sub-groups, known as most-at-risk or key populations, tend to have consistently higher rates of new and existing infections than the general population [1], [3]. A growing body of evidence from Uganda and other sub-Saharan African countries suggests that fisher folk communities (FFC) appear to have a much higher burden of HIV-1 infection than respective general populations [4]–[11]. This may not be surprising given that the first cases of HIV in Uganda were identified from a fishing community in Rakai district in 1982 [12] and since then FFC in East Africa have been disproportionately affected by the HIV epidemic [13]–[16]. However, most studies have reported findings on HIV prevalence in FFC but data on incidence are still very limited. HIV prevalence levels between 27–29% in Ugandan FFC and 16–25% among Kenyan FFC [4]–[6], [9], [10], [17], [18] and an incidence rate of 4.9/100 person-years among Ugandan FFC prescreened and identified as being at high-risk of HIV infection [4], [11] have been reported. These figures are 3–4 times higher than respective national averages. Currently in Uganda, groups that have been identified as key populations include commercial sex workers, uniformed services, fishing communities, truck drivers and MSMs [18] but only commercial sex workers and long distance truck drivers have been well characterized [19]–[21]. However, emerging data on FFC and men having sex with men suggest that these groups too may be key populations [4], [5], [11], [22]. Unlike general populations, fishing communities tend to be socially marginalized and often stigmatized. They characterized by a high presence of bars, lodges and entertainment halls, transactional sex (sex for money and sex for fish) activities, high alcohol consumption and multiple sexual partnerships [4], [5], [8], [9], [11], [13], [15], [23], [24], but very limited access to health services. Fisherfolk, especially fishermen and fish traders, tend to be mobile or migratory moving between islands and landing sites in search for better fish yields [7], [25]. The mobile lifestyle is believed to contribute to risk behaviours in part due to absence social structures that could constrain risk taking such as spousal and family social audit [13], [24], [26]. Among fishermen, the daily risk of drowning with immediate death appears to be a bigger concern than the risk of contracting a chronic and nowadays not necessarily fatal infection with HIV [4].

More empirical data on HIV incidence, risk factors and drivers of HIV infection are urgently needed to determine whether FFC are indeed a key HIV population and to facilitate the design and implementation of appropriate prevention and control strategies in FFC and groups with similar characteristics. Sexual inter-mixing and interactions between persons in key populations and the general population may dilute the gains thus far attained in reducing HIV in latter. Additionally, the fishing business provides food and economic survival to a large number of people in East and Southern Africa and contributes to national economic development. In Uganda alone, it is estimated that FFC constitutes about three million people whose fishing business contributes over 6% to the national gross domestic product [18]. It is therefore critical to determine the burden of the HIV epidemic in FFC and to provide timely and appropriate control measures. We conducted a community-based cohort study in a randomly selected population representative sample in 8 fishing communities to determine the HIV-1 incidence and associated risk factors in the general FFC population around Lake Victoria, Uganda.

## Methods

### Study sites definition

A fishing community was defined as a group of persons living in a village or trading center that is adjacent to lake landing site where main economic activities and livelihood are derived directly or indirectly from fishing activities. Inhabitants of these communities are diverse and usually include fishers (boat crew), boat owners, boat makers and repairers, fish processors and traders, shop keepers, and owners and workers of bars/restaurants/lodges. These communities are typically densely populated with wooden temporary buildings that are densely concentrated in small spaces especially at landing sites and their proximal areas [17].

### Study design and Procedures

A community-based cohort study was conducted in 8 fishing communities (1 lakeshore and 7 islands) in 3 Uganda districts of Wakiso, Mukono, and Kalangala. Study procedures have been previously described [5] but briefly, we conducted a community-wide household enumeration census in each community after which a proportion to size random sample of 2200 participants aged 18–49 years was selected using Stata 12 (StataCorp, College Station, TX) software. Of the 2200 selected, 2191 provided written informed consent and were interviewed in privacy by same sex interviewers at baseline and 1685 at the 12 months post-baseline visit. Data on socio-demographic characteristics and risky behaviors (including number of partners, new partners, condom use, use of alcohol and illicit drugs) were collected using semi-structured questionnaires. Participants were asked if they drank any alcoholic drink and if they responded in affirmation, they were further asked the frequency of consumption. Assessments were done for 3 and 12 months preceding the date of interview. Venous blood samples were collected for HIV-1 serological testing and participants got voluntary counseling and testing from certified HIV counselors. Participants were encouraged to share their HIV results with their sexual partners but no involuntary disclosure of HIV results to third parties was done as per the Ugandan Ministry of Health AIDS Control Program policy on HIV testing [27]. HIV infected participants were referred to HIV/AIDS care centres for further management and encouraged to seek care. HIV prevention services including health education, counseling, treatment of sexually transmitted infections (STIs) and voluntary medical male circumcision were provided to community members (participants and non-participants) at no cost. Institutional Review Board approvals were obtained from the Uganda Virus Research Institute's Science and Ethics Committee (UVRI SEC) and the Uganda National Council for Science and Technology (UNCST). All participants were enrolled in the study after providing written informed consent.

### Laboratory testing

HIV-1 serology was determined by rapid HIV tests performed in the community by certified laboratory technologists and EIA confirmation in the laboratory at Uganda Virus Research Institute. In the rapid HIV testing algorithm blood samples were first tested with Determine HIV assay (Alere Medical Co., Ltd., Chiba, Japan), and if negative, results were reported as negative. Determine positive samples were then tested with HIV 1/2 Stat-Pak assay (Chembio Diagnostic Systems, Inc. Medford, NY, USA), and if positive too, results were reported as positive. But if negative on Stat-Pak, Uni-Gold HIV test (Trinity Biotech plc, Bray, Ireland) was used as a tie-breaker. All positive rapid results were confirmed using 2 parallel enzymelinked immunosorbent assay (EIA) tests: Vironostika (HIV Uni-Form II plus 0 microelisa system, Biomerieux, SA, Marcy l'Etoile, France); and Murex HIV-1.2.O (Diasorin S.P.A, Dartford, United Kingdom). Concordinant EIA positives were taken as positive but discordant EIA results were comfirmed using HIV RNA PCR (COBAS AmpliPrep/COBAS TaqMan HIV-1 Test, v2.0 from Roche Molecular Diagnostics, Pleasanton, CA, USA).

### Statistical Analysis

Participants' characteristics were summarised and compared using *t*-tests for continuous variables and chi-square and Fisher Exact tests for categorical variables. Bivariate analyses were used to estimated unadjusted (crude) associations between outcome variables and potential predictors. Adjusted associations were estimated using mutlivariable regression models. All models were constructed using stepwise logical model building method (purposeful selection of covariates) [28] and the most persimonious model was selected as the final one. Covariates were selected for inclusion in multivariable models based on a bivariate statistical significance at an alpha (α) of <0.15 and biological plausibility (clinical and intuitive relationship to outcome variable). To account for potential correlation at household level (where more than 1 participant from a gvien household were selected) we used the empirical variance estimator to estimate robust standard errors [29]. All statistical analyses were performed using Stata 12 (StataCorp, College Station, TX) software.

The main outcome was incident HIV infection among previously uninfected individuals at baseline. HIV seroconversion was estimated to have occurred at the midpoint between the last negative and first positive serologic tests, approximately 12 months apart. Person years at-risk (pyar) were calculated as: (date of last HIV seronegative result, or estimated date of HIV seroconversion minus date of enrollment) divided by 365.25. HIV incidence rate per 100 pyar was calculated as number of events of seroconversion divided by pyar, multiplied by 100. Adjusted incidence rate ratios (Adj.IRR) of HIV acquisition with corresponding 95% confidence intervals were estimated by multivariable Poisson regression using the natural logarithm of pyar as the offset term. The final model on HIV acquisition included sex, age, religion, marital status, new sexual partners in past 12 months, and frequency of alcohol drinking. Alcohol consumption was defined as occassional if the participants reported consumption at least once a week or less, and regular if they drank daily or at least 3 days every week. Covariates that dropped from the final model include ethnicity/tribe, duration of stay in fishing communities, occupation, marital status, condom use, male circumcision status, and use of marijuana.

## Results

Of the 2191 participants enrolled at baseline, 1685 were interviewed during the 12 months post-baseline visit giving a follow up rate of 76.9% (Table 1). Follow up rates did not statistically differ by sex, education status and religion but were significantly lower among those aged 18–24 years, non-Baganda, never married, bar/lodge/restaurant workers, those with less than one year's stay in fishing communities, and HIV positives at baseline. Among the 1288 participants included in incidence analysis, the mean (SD) and median (IQR) age in years were 29.7 (7.5) and 29 (24–35) respectively. Fifty four percent (54.1%) were males, 20.4% Moslems, 40.2% Protestants/Evangelical, and 39.4% Catholics. Only 35.9% had attained post primary education, 49.5% had been residing in their community for less than 5 years, and 69.2% were married. Fifty percent (50.3%) were involved in fishing and fishing related activities, 10.5% in small scale businesses, 5.8% were farmers, and 9.5% were bar/lodge/restaurant workers (not shown).

**Table 1:** Baseline Socio-demographic Characteristics of Study Population by Enrolment Status.

	Enrolment	Follow-up Status	
	Enrolled No. (%)	Followed No. (%)	Not Followed No. (%)
**All Participants**	2191 (99.5)	1685 (76.9)	506 (23.1)
**Sex**			
Male	1106 (50.5)	865 (78.2)	241 (21.8)
Female	1085 (49.5)	820 (75.6)	265 (24.4)
**Age at enrolment (years)**			
18–24	616 (28.1)	429 (69.6)	187 (30.4)†
25–29	566 (25.8)	434 (76.7)	132 (23.3)
30–39	733 (33.5)	591 (80.6)	142 (19.8)
40–49	276 (12.6)	231 (83.7)	45 (16.3)
**Highest Education level** *			
None	186 (8.5)	138 (74.2)	48 (25.8)
Primary	1294 (59.1)	987 (76.3)	307 (23.7)
Post primary	708 (32.4)	557 (78.7)	151 (21.3)
**Religion**			
Roman Catholic	890 (40.6)	681 (76.5)	209 (23.5)
Protestant/Anglican	600 (27.4)	451 (75.2)	149 (24.8)
Moslem	421 (19.2)	329 (78.2)	92 (21.8)
Pentecostal/Evangelical	197 (9.0)	160 (81.2)	37 (18.8)
Other¶	83 (3.8)	64 (77.1)	19 (22.9)
**Ethnicity/tribe**			
Non-Muganda	1197 (54.6)	881 (73.6)	316 (26.4)†
Muganda	994 (45.4)	804 (80.9)	190 (19.1)
**Occupation**			
Fishing/Fishing related	1038 (47.4)	817 (78.7)	221 (21.3)
Trade/Business	223 (10.2)	176 (78.9)	47 (21.9)
Bar/Lodge/Restaurant	257 (11.7)	170 (66.2)	87 (33.8)†
Farming	130 (5.9)	111 (85.4)	19 (14.6)
Others 	353 (16.1)	272 (77.0)	81 (22.9)
Housewife	190 (8.7)	139 (73.2)	51 (26.8)
**Marital status**			
Never married	340 (15.5)	233 (68.5)	107 (31.5)†
Not currently married	505 (23.1)	376 (74.5)	129 (25.5)
Married monogamous	923 (42.1)	743 (80.5)	180 (19.5)
Married polygamous	423 (19.3)	333 (78.7)	90 (21.3)
**Duration in community** (years)			
Less than 1	394 (17.9)	213 (54.1)	181 (45.9)†
1 to 4	823 (37.6)	609 (74.0)	214 (26.0)
5 to 10	668 (30.5)	589 (88.0)	80 (12.0)
More than 10	305 (13.9)	274 (89.8)	31 (10.2)
**Alcohol consumption**			
No	1031 (47.1)	799 (77.5)	232 (22.5)
Yes	1160 (52.9)	886 (76.4)	274 (23.6)
**Use of Marijuana**			
No	1889 (86.2)	1460 (77.3)	429 (22.7)
Yes	302 (13.8)	225 (74.5)	77 (25.5)
**HIV status (Baseline)**			
Positive	584 (26.6)	396 (67.8)	188 (32.2)†
Negative	1607 (73.3)	1289 (80.2)	318 (19.8)

*3 missing education,

¶ Seventh Day Advent/Traditionist,


Construction/Mechanic/Government/Clerical,

† P<0.05.

### HIV incidence rate and associated factors

There were 48 incident HIV infections among 1288 participants followed over 1416.8 person years at risk (pyar) yielding an overall cumulative incidence of 3.72% (95% CI, 2.74–4.94) and an incidence rate of 3.39/100 pyar (95% CI, 2.55–4.49). Table 2 shows absolute incidence rates individual and combined by socio-demographic characteristics and risky behaviours. The absolute HIV incidence rate (AR) was 3.40 (2.31–4.99) in men, 3.37 (2.22–5.13) in women, 3.68 (2.22–6.11) among those aged 18–24 years, 4.77 (2.96–7.67) in 25–29 years, and 2.45 (1.50–4.00) in those aged 30 years or more. The AR was 5.44 (3.80–7.78) among Roman Catholics, 2.09 (1.18–3.67) in Protestants/Evangelicals and 2.06 (0.93–4.60) in Moslems. Unmarried participants had an AR of 5.04/100 pyar while it was 2.65/100 pyar among the married ones [not shown]. High ARs were observed among Roman Catholics [5.44 (95% CI; 3.80–7.78)], those involved in fishing-related activities [5.46 (95% CI; 3.02–9.86)], participants previously married but not married at the time of the study [5.62 (95% CI; 3.44–9.18)], those who reported 2 or more new sexual partners in past 12 months [5.67 (95% CI; 3.14–10.23)], and regular alcohol drinkers [6.44 (95% CI; 4.38–9.45)]. But the highest incidence was observed among alcohol drinkers aged 25–29 years [7.67 (95% CI; 4.62–12.7)]. It is noteworthy that involvement in fishing per se did not increase the absolute risk of HIV as long as one was young (less than 30 years) and consumed alcohol.

**Table 2:** HIV Incidence Rate by Socio-demographic Characteristics and Risky Behaviours.

Characteristic	Incidence/100		
	Cases	PYAR¶	Rate (95% CI)
**All Participants**	48	1416.8	3.39 (2.55–4.49)
**INDIVIDUAL VARIABLES**			
**Sex**			
Male	26	765.2	3.40 (2.31–4.99)
Female	22	651.5	3.37 (2.22–5.13)
**Age at enrolment (years)**			
30+	16	652.8	2.45 (1.50–4.00)
25–29	17	356.5	4.77 (2.96–7.67)
18–24	15	407.4	3.68 (2.22–6.11)
**Religion**			
Moslem	6	290.5	2.06 (0.93–4.60)
Protestant/Evangelical	12	575.0	2.09 (1.18–3.67)
Roman Catholic	30	551.3	5.44 (3.80–7.78)
**Ethnicity/tribe**			
Muganda	17	675.5	2.52 (1.56–4.05)
Non-Muganda	31	741.2	4.18 (2.94–5.95)
**Occupation**			
Trade/Business	2	153.0	1.31 (0.33–5.22)
Fishing	16	504.9	3.17 (1.94–5.17)
Fishing related activities†	11	201.4	5.46 (3.02–9.86)
Bar/Lodge/Restaurant	5	136.7	3.66 (1.52–8.79)
Farming	3	79.8	3.76 (1.21–11.6)
Housewife	3	110.6	2.71 (0.87–8.41)
Others††	8	230.3	3.47 (1.74–6.95)
**Duration in community at enrolment** (years)			
5+	21	713.4	2.94 (1.91–4.51)
2–4	11	366.1	3.00 (1.66–5.42)
Less than 2	16	337.2	4.75 (2.91–7.74)
**Marital status**			
Married monogamous	18	632.8	2.84 (1.79–4.51)
Married polygamous	7	260.2	2.84 (1.79–4.51)
Not currently married	16	284.5	5.62 (3.44–9.18)
Never married	7	239.2	2.93 (1.39–6.14)
**New sex partners in past 12 months**			
None	22	827.5	2.66 (1.75–4.04)
1	7	251.9	2.78 (1.32–5.82)
2+	11	194.1	5.67 (3.14–10.23)
**Condom use in past 12 months**			
Always	4	191.0	2.09 (0.78–5.58)
Inconsistent	15	319.4	4.69 (2.83–7.79)
No use	29	769.7	3.77 (2.62–5.42)
**Circumcised** (men only)			
Yes	8	355.2	2.25 (1.13–4.50)
No	18	408.5	4.40 (2.77–6.99)
**Frequency of Alcohol consumption**			
No	10	668.4	1.50 (0.80–2.78)
Occasional	12	344.5	3.48 (1.98–6.13)
Regular	26	403.9	6.44 (4.38–9.45)
**Use of Marijuana**			
No	44	1299.3	3.39 (2.52–4.55)
Yes	4	104.5	3.83 (1.44–10.20)
**COMBINED VARIABLES**			
**Age and fishing**			
30+ and involved in fishing	11	336.9	3.26 (1.80–5.90)
25–29 and involved in fishing	9	195.4	4.61 (2.40–8.85)
18–24 and involved in fishing	7	174.1	4.02 (1.92–8.43)
**Age and alcohol drinking**			
30+ and drinks alcohol	12	359.0	3.34 (1.89–5.89)
25–29 and drinks alcohol	15	195.5	7.67 (4.62–12.7)
18–24 and drinks alcohol	11	193.8	5.67 (3.14–10.2)
**Age, fishing and alcohol drinking**			
30+, fishing and alcohol use	7	196.1	3.57 (1.70–7.49)
25–29, fishing and alcohol use	8	118.1	6.77 (3.39–13.5)
18–24, fishing and alcohol use	4	80.2	4.99 (1.87–13.3)

¶ PYAR-person years at risk,

† Fishmongers, fish processing, boat maker/owner,

†† Construction/Mechanic/Government/Clerical.

Table 3 shows unadjusted and adjusted incident rate ratios (IRRs) of HIV and associated 95% confidence intervals. At bivariate analysis, the unadjusted (crude) risk of HIV infection was 2.6 times higher among Roman Catholics relative to Moslems (crude IRR = 2.63, 95% CI, 1.10–6.33), and 2 times higher among participants with 2 or more new sex partners in past 12 months compared to those with none (crude IRR = 2.13, 95% CI, 1.03–4.39). Compared to those who reported no alcohol consumption, the risk of HIV infection was twice as high among occasional drinkers and four times higher among regular drinkers - IRR = 2.33(95% CI, 1.01–5.39) and 4.30 (95% CI, 2.07–8.92), respectively. The unadjusted risk was 2 times higher among those aged 25–29 years compared to those aged 30 or more years but the difference was of borderline statistical significance [IRR = 1.94 (95% CI, 0.98–3.85)].

**Table 3:** Unadjusted and Adjusted Incidence Rate Ratios of HIV Acquisition and Associated Factors.

Characteristic	Incidence Rate Ratio (95% CI)		
	Unadjusted	Adjusted	P-value
**Sex**			
Male	1 (Reference)	1 (Ref)	
Female	0.99 (0.56–1.75)	1.15 (0.58–2.29)	0.68
**Age** (years)			
30+	1 (Ref)	1 (Ref)	
25–29	1.94 (0.98–3.85)	3.36 (1.48–7.65)	0.004
18–24	1.50 (0.74–3.04)	2.65 (1.05–6.70)	0.039
**Religion**			
Moslem	1 (Ref)	1 (Ref)	
Protestant/Evangelical	1.01 (0.38–2.69)	0.78 (0.26–2.27)	0.645
Roman Catholic	2.63 (1.10–6.33)	1.65 (0.62–4.38)	0.317
**Ethnicity/tribe**			
Muganda	1 (Ref)	-	
Non-Muganda	1.66 (0.92–3.00)	-	
**Occupation**			
Trade/Business	1 (Ref)	-	
Fishing	2.42 (0.56–10.5)	-	
Fishing related activities^†^	4.18 (0.93–18.8)	-	
Bar/Lodge/Restaurant	2.80 (0.54–14.4)	-	
Farming	2.88 (0.48–17.2)	-	
Housewife	2.07 (0.35–12.4)	-	
Others^††^	2.66 (0.56–12.5)	-	
**Duration in community at enrolment** (years)			
5+	1 (Ref)	-	
2–4	1.02 (0.49–2.12)	-	
Less than 2	1.61 (0.84–3.09)	-	
**Marital status**			
Married monogamous	1 (Ref)	-	
Married polygamous	0.94 (0.39–2.26)	1.20 (0.46–3.11)	0.712
Not currently married	1.98 (1.01–3.87)	2.06 (0.92–4.61)	0.079
Never married	1.03 (0.43–2.46)	0.84 (0.28–2.50)	0.749
**New sex partners in past 12 months**			
None	1 (Ref)	1 (Ref)	
1	1.04 (0.45–2.45)	0.89 (0.37–2.14)	0.793
2+	2.13 (1.03–4.39)	1.31 (0.58–2.99)	0.513
**Condom use in past 12 months**			
Always	1 (Ref)	-	
Inconsistent	2.24 (0.74–6.75)	-	
No use	1.80 (0.63–5.11)	-	
**Circumcised** (men only)			
Yes	1 (Ref)	-	
No	1.96 (0.85–4.50)	-	
**Frequency of Alcohol consumption**			
No	1 (Ref)	1 (Ref)	
Occasional	2.33 (1.01–5.39)	3.18 (1.18–8.57)	0.022
Regular	4.30 (2.07–8.92)	4.93 (1.91–12.8)	0.001
**Use of Marijuana**			
No	1 (Ref)	-	
Yes	1.13 (0.41–3.15)	-	

At multivariable analysis, the risk of HIV infection was statistically significantly associated with age and alcohol consumption, and there was a borderline association with those previously married but not currently married. Compared to participants aged 30 or more years, the adjusted HIV incidence rate ratios were 3.36 (95% CI, 1.48–7.65) and 2.65 (95 CI, 1.05–6.70) for participants aged 25–29 years and 18–24 years respectively.

The risk of HIV infection increased with increasing frequency of alcohol consumption. Compared to non-drinkers, the adjusted risk was 3 times higher in occasional drinkers and 5 times higher in regular drinkers - adj.IRR = 3.18 (95% CI, 1.18–8.57) and 4.93 (95% CI, 1.91–12.8), respectively. The effect of alcohol on HIV risk was more pronounced in the age group of 25–29 years (Table 4). Without considering frequency of alcohol consumption, the incident rate ratio of HIV acquisition among alcohol drinkers compared to non-drinkers was 2.45 (95% CI, 0.79–7.61), 6.18 (95% CI, 1.41–27.0), and 3.03 (95% CI, 0.96–9.51) for age years 30+, 25–29 and 18–24 respectively. Compared to non-drinkers, the risk of HIV increased with increasing frequency of alcohol consumption in each age group. Across age groups, the risk was higher among those aged 25–29 years, followed by 18–24 years and lowest among those aged 30 or more years for both occasional and regular drinkers. The most pronounced effect of alcohol on HIV risk was observed among regular drinkers aged 25–29 years, IRR = 8.44 (95% CI, 1.85–38.5).

**Table 4:** Frequency of alcohol drinking on HIV-1 incidence by age group.

Characteristic	IRR (95% CI) by Age group		
	30+	25–29	18–24
**Alcohol drinking**			
No	1 (Ref)	1 (Ref)	1 (Ref)
Yes	2.45	6.18	3.03
	(0.79–7.61), *p* = 0.12	(1.41–27.0), *p* = 0.016	(0.96–9.51), *p* = 0.058
**Frequency of alcohol drinking**			
No	1 (Ref)	1 (Ref)	1 (Ref)
Occasional	1.54	4.02	2.10
	(0.34–6.89), *p* = 0.57	(0.78–20.7), *p* = 0.096	(0.53–8.41), *p* = 0.293
Regular	3.06	8.44	4.04
	(0.94–9.93), *p* = 0.063	(1.85–38.5), *p* = 0.006	(1.18–13.8), *p* = 0.026

Although the risk of HIV infection was significantly higher among Catholics than Moslems at bivariate analysis [crude IRR = 2.63 (95% CI; 1.10–6.33)], the association lost statistical significance after multivariable adjustment [adj. IRR = 1.65 (95% CI; 0.62–4.38)]. Nevertheless, we assessed whether the association between incident HIV infection and religion was confounded by alcohol consumption and male circumcision. Alcohol consumption was higher among Catholics (51.0%), than Protestants (41.5%) and Moslems (27.5%) [trend *p*<0.0001]. As expected male circumcision higher among Moslems (96.7%) than Protestants (32.5%) and Catholics (28.9%) [trend *p*<0.0001]. For all the religious groups, the absolute incidence of HIV was higher among those who reported alcohol drinking (any frequency) than their counterparts who reported no alcohol consumption in past 12 months; rates were 3.11 (95% CI; 1.00–9.67) versus 1.54 (95% CI; 0.49–4.79) among Moslems, 3.47 (95% CI; 1.87–6.45) versus 0.70 (95% CI; 0.17–2.79) in Protestants/Evangelicals, and 6.86 (95% CI; 4.64–10.16) versus 2.67 (95% CI; 1.11–6.42) for Catholics. When Catholics and Moslems who drink alcohol were compared, adjusting for circumcision, the IRR of HIV acquisition was 2.56 (95% CI; 0.29–22.54), *p* = 0.398. Results were similar when non-alcohol drinkers were compared - adj.IRR = 2.44 (95% CI; 0.22–26.94), *p* = 0.466 [Not shown].

## Discussion

In a general population cohort study in fishing communities around Lake Victoria, Uganda, we found an overall HIV-1 incidence rate of 3.39/100 pyar (95% CI, 2.55–4.49) with the highest incidence of 7.67 (95% CI; 4.62–12.7) observed among alcohol drinkers aged 25–29 years. The risk of HIV infection was mainly associated with young age (less than 30 years) and alcohol consumption.

The incidence of HIV that we observed in this general FFC population study was lower than that found in fisherfolk that were screened for high risk behaviour in a previous study that was conducted in Entebbe and Masaka communities (3.4 /100 vs 4.9/100 pyar, *p* = 0.059) [11]. However, the general FFC population rate in this study conducted in Entebbe site communities was not different from that observed among high risk FFC from the same side of the lake (3.4/100 vs 3.8/100 pyar, *p* = 0.588) [11]. Our data seems to suggest that for adult sexually active persons, living in a fishing community is generally associated with increased risk for HIV infection. But strong conclusions on this observation can be better made from studies that involve both fishing and non fishing communities in which direct comparisons of HIV rates could be done. However, findings from Rakai district, Southwestern Uganda, indicate that HIV rates are highest in lake shore communities, followed by adjacent communities and are lowest in agrarian non fishing communities (pc Dr.David Serwadda). Our finding of similar risk of HIV infection between general population of fisherfolk and those screened for high risk implies that HIV prevention and control programs as well as intervention studies should target the fisherfolk community as a whole without pre-screening for “high risk”. It is noteworthy that the overall risk of HIV infection of 3.39/100 pyar that we found among general population of fishing communities is about 4 times higher than the estimated national incidence among adults in general population in Uganda [30]. However, the risk among persons in fishing communities who are considered to be “high risk” is about 5–8 times higher than the estimated national rate among adults. This underscores the need for urgent interventions to prevent and control the spread of HIV in fishing communities which tend to be socially margnalized and under served. Furthermore, there is need for more studies in fishing communities to concretize the evidence that these communities are key HIV populations. With such high risks of HIV infection in fishing communities, sexual inter-mixing and interactions between persons in these communities and the general populations may lead to an upsurge of HIV incidence in general population.

We found a higher rate of HIV infection among young people (aged 18–29 years) which is consistent with an earlier study [11] but differs from the observation of higher rates of infection among older people in the general population in Uganda [1]. This difference highlights the potential variations in HIV risk profiles (risk factors and drivers) between general population and key populations within the same regional/geographical HIV sub-epidemic.

Alcohol consumption was a very strong predictor of risk of HIV infection in this study; regular drinkers were 5 times more likely to get infected with HIV compared to non-drinkers. This finding too is consistent with previous studies among FFC in Uganda that reported higher risk of HIV infection among regular alcohol drinkers and a strong correlation between alcohol consumption and risky behaviours such as having multiple sexual partners, sex with non-regular partner and transactional sex [4], [11], [31]. In this study we explored the association between HIV incidence and alcohol consumption stratified by religion and found that in all religious groups (Moslems, Protestants/Evangelicals, and Catholics) the absolute incidence of HIV infection was 2–5 times higher among alcohol drinkers compared to non-drinkers. Despite the 97% circumcision level among Moslems, those who drank alcohol had a 2 times absolute risk of HIV infection relative to their non-drinking counterparts. The increased risk of HIV infection in Moslems who drink alcohol raises the question whether alcohol consumption might diminish the impact of circumcision in preventing HIV spread but this hypothesis warrants further studying. In general, the problem of alcohol consumption in fishing communities needs to be addressed not only due to its strong association with risk of HIV infection but also in its own entity as a psychosocial problem. Interventions to reduce hazardous alcohol use have been shown to lower unprotected sex [32] which might result in reduced risk of HIV acquisition and transmission.

The strengths of this study include; 1) it was conducted in a general FFC population that was randomly selected which enabled estimation of population-wide HIV rates, 2) study communities included islands and lakeshores unlike previous studies that were conducted exclusively in lakeshore communities, 3) none of the study communities had been involved in previous epidemiological studies thus the observed rates are unlikely to have been influenced by prior studies and their related interventions, and 4) study communities came from 3 Ugandan districts along L.Victoria which ensured a wide geographical representation. Nonetheless, this study had some limitations. First, inability to assess sexually transmitted infections (STIs) which are known co-factors for HIV infection and hence important to adjust for in estimation of HIV rates. Second, lack of inclusion of non fishing communities which would have facilitated direct comparisons of HIV rates between fishing and non fishing communities. Lastly, the assessment of alcohol consumption and illicit drug use did not use conventionally standardized questions.
